# Sources of exposure and risk among employees infected with severe acute respiratory coronavirus virus 2 (SARS-CoV-2) in a large, urban, tertiary-care hospital in the United States

**DOI:** 10.1017/ash.2022.366

**Published:** 2023-01-30

**Authors:** Cassidy Boomsma, Dina Poplausky, Jacob M. Jasper, MacKenzie Clark MacRae, Alice M. Tang, Elena Byhoff, Alysse G. Wurcel, Shira Doron, Ramnath Subbaraman

**Affiliations:** 1 Tufts University School of Medicine, Boston, Massachusetts; 2 Department of Public Health and Community Medicine and Center for Global Public Health, Tufts University School of Medicine, Boston, Massachusetts; 3 Division of Geographic Medicine and Infectious Diseases, Tufts Medical Center, Boston, Massachusetts; 4 Department of Medicine, Tufts Medical Center, Boston, Massachusetts

## Abstract

**Objective:**

Hospital employees are at risk of SARS-CoV-2 infection through transmission in 3 settings: (1) the community, (2) within the hospital from patient care, and (3) within the hospital from other employees. We evaluated probable sources of infection among hospital employees based on reported exposures before infection.

**Design:**

A structured survey was distributed to participants to evaluate presumed COVID-19 exposures (ie, close contacts with people with known or probable COVID-19) and mask usage. Participants were stratified into high, medium, low, and unknown risk categories based on exposure characteristics and personal protective equipment.

**Setting:**

Tertiary-care hospital in Boston, Massachusetts.

**Participants:**

Hospital employees with a positive SARS-CoV-2 PCR test result between March 2020 and January 2021. During this period, 573 employees tested positive, of whom 187 (31.5%) participated.

**Results:**

We did not detect a statistically significant difference in the proportion of employees who reported any exposure (ie, close contacts at any risk level) in the community compared with any exposure in the hospital, from either patients or employees. In total, 131 participants (70.0%) reported no known high-risk exposure (ie, unmasked close contacts) in any setting. Among those who could identify a high-risk exposure, employees were more likely to have had a high-risk exposure in the community than in both hospital settings combined (odds ratio, 1.89; *P* = .03).

**Conclusions:**

Hospital employees experienced exposure risks in both community and hospital settings. Most employees were unable to identify high-risk exposures prior to infection. When respondents identified high-risk exposures, they were more likely to have occurred in the community.

The coronavirus disease 2019 (COVID-19) pandemic has put strain on healthcare workers worldwide since it emerged in December 2019 and was declared a pandemic by the World Health Organization in March 2020.^1^ Even after the advent of vaccination against SARS-CoV-2, healthcare workers have been at increased risk of infection due to their risk of occupational exposure through patients and coworkers.^2^

Considerable research has been conducted to better understand the potential increased risk of infection for people working in the hospital, but questions remain.^3^ Many studies have investigated risk factors for healthcare worker SARS-CoV-2 infection through interviews, questionnaires, assessing seroprevalence, and screening asymptomatic individuals. Results of these studies have been variable, with some finding high-risk exposure outside the workplace as the strongest risk factor^4–8^ and others finding that exposure to patients increases the probability of a positive SARS-CoV-2 test.^9–13^ Staff-to-staff transmission also represents a significant driver of employee infection.^12,14–16^ Prior studies have been limited in that most investigated patient-facing job roles^17–19^ as potential risk factors and relatively few considered the potential contribution of transmission from coworkers.^20–22^ Additionally, mask use was investigated in some studies^9,10,14,17^ but not in others.^14,21,23^ Universal masking of both healthcare workers and patients has been associated with a lower rate of SARS-CoV-2 positivity among healthcare workers.^24^

Recognizing the gaps in the literature, we devised a study to evaluate exposures among all hospital employees, not just those who were patient facing. We evaluated exposures in the hospital and in the community among employees who tested positive for SARS-CoV-2 by nasal polymerase chain reaction (PCR) testing at a large, urban, tertiary-care hospital in the United States. We sought to understand exposures, and we evaluated mask usage and breaches during hospital and community interactions to stratify risk as well as potential employee-to-employee transmission.

## Methods

### Study design

We used a structured survey to assess exposures among hospital employees infected with COVID-19 between March 1, 2020, and January 15, 2021. “Employees” refers to all individuals who work within the hospital premises, whether they are patient facing or non–patient facing, or full-time employees or contract workers. Employees who tested positive for SARS-CoV-2 by nasal PCR testing were asked to complete an anonymous survey detailing potential exposures in the 2 weeks prior to the start of their symptoms or prior to their positive test result. Exposures were considered in 3 different settings: (1) in the community (ie, any exposures outside of the hospital), (2) in the hospital from contact with patients, and (3) in the hospital from contact with other employees.

### Study setting

This study was conducted at Tufts Medical Center (TMC), a tertiary-care academic medical center in Boston, Massachusetts, with ∼7,000 employees. Universal masking was implemented on March 27, 2020, for employees, patients, and visitors, but notably was not consistently enforced for inpatients. From March to June 2020, by hospital policy and consistent with CDC guidelines during times of severe shortage,^25^ N95 masks were only recommended for use during interactions with COVID-19 patients in intensive care units or during or after aerosol-generating procedures. Universal use of N95 masks for all patients with confirmed or suspected COVID-19 in the hospital was implemented on June 11, 2020.

### Study participants

Study participants were TMC employees who had a positive SARS-CoV-2 PCR test result between March 1, 2020, and January 15, 2021, either according to the employee’s self-report or as documented by the TMC employee health department. The employee health department maintained a record of employees who tested positive through the hospital’s testing facility or from an outside facility per self-report.

### Survey

A structured questionnaire was fielded to participants to evaluate COVID-19 exposures and personal protective equipment (PPE) in community and hospital settings. Close contact was defined as being within 6 feet (2 meters) of someone with confirmed or probable COVID-19 for at least 15 minutes or having direct contact with their secretions (saliva, getting coughed on, etc), regardless of whether PPE was worn. Participants described mask usage in each reported exposure, specifically whether both individuals were masked, one individual was masked, or neither individual was masked. When describing contact with TMC patients, participants answered questions regarding eye protection, gown, and glove usage, and participation in aerosol-generating procedures. They were also asked to explain their perceived source of infection using open-ended free text. Participants provided demographic data and COVID-19 symptoms.

### Distribution of survey

The survey was distributed in 2 waves. The first wave of surveys was distributed in July and August 2020 as part of another TMC study evaluating disparities in SARS-CoV-2 testing among employees.^26^ This survey was offered in 5 different languages: English, Spanish, Haitian Creole, Portuguese, Cantonese, and Mandarin, with online and paper versions. This initial survey wave involved outreach to all TMC employees, regardless of COVID-19 status. Employees who self-reported testing positive for SARS-CoV-2 were asked to answer additional survey questions. The second survey wave was distributed in English between March 1, 2021, and June 10, 2021, to all SARS-CoV-2-positive employees who had not already participated, as identified through TMC employee health department records. Only the first wave of participants were compensated with a $10 gift card. Participants were recruited by e-mail, and our team followed up with a single telephone call to those who did not initially respond to encourage participation.

## Risk classification

Participants were first grouped into categories based on their exposures in each of the 3 settings: community, in the hospital via patient, or in the hospital via another employee. Notably, participants could report exposures in multiple settings (ie community and/or patient and/or employee), and be grouped in multiple risk categories. These categories included “no exposure” (ie, no known close contact with a SARS-CoV-2-infected individual in that setting) or subcategories among those who had exposure (ie, known close contact with a SARS-CoV-2-infected individual). These subcategories were “exposure with both masked,” (ie, close contact but with the study participant and their contact masked for the duration of the encounter), “exposure with one masked” (ie, either the study participant or their contact was masked, but not both), “exposure with no masking,” (ie, neither the study participant nor their contact was masked), or “exposure with masking responses missing” (ie, participant reported an exposure but did not answer masking questions). Likewise, participants were grouped into an “exposure responses missing” category, if they did not answer exposure questions.

**Fig. 1.**
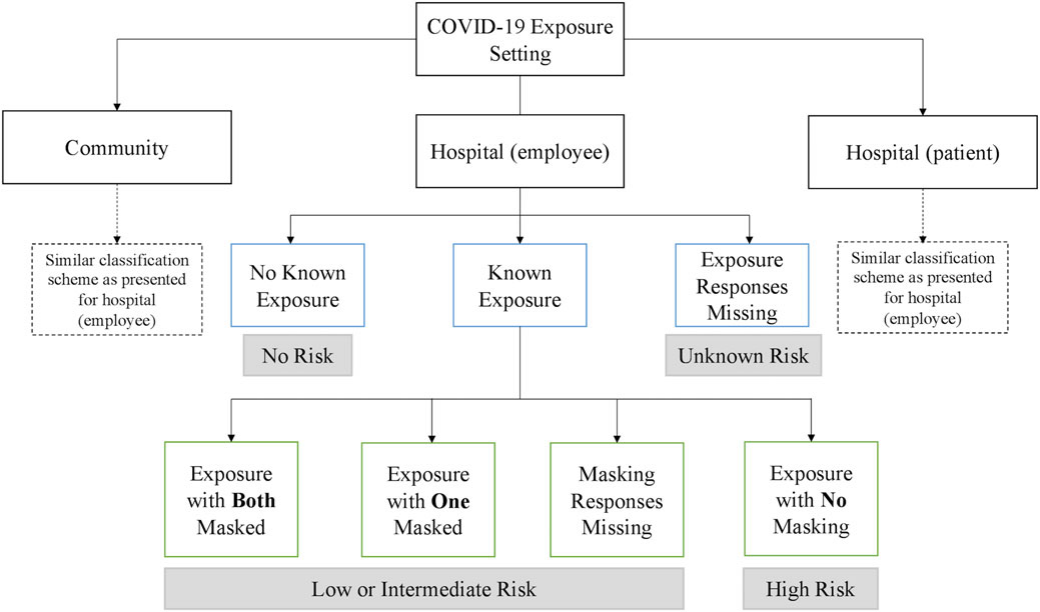
Approach to classifying the level of SARS-COV-2 exposure risk. Notably, participants could report exposures in multiple settings (ie, community and/or hospital (employee), and/or hospital (patient)), and in that case would be counted in multiple exposure groups. The classification scheme is presented for the hospital (employee) setting as an example; a similar approach to classification was used for the other 2 settings. Classification of risk in the patient setting assumed that all patients were unmasked and that aerosol-generating procedures conducted without a N95 mask (even if a non-N95 medical-grade mask was used) constituted a high-risk exposure. While providing patient care, masked exposures without eye protection, or with body contact while not wearing a gown and gloves, were also classified as low or intermediate risk. If a participant did not answer survey questions regarding exposures or masking, they were grouped into “exposure responses missing” and “masking responses missing” respectively. See supplementary text for more information about risk classification.

Participants were then classified into risk categories based on their reported exposure status in each setting (Fig. 1). These risk categories included “no risk,” “low or intermediate risk,” or “high risk.” “No risk” was defined as situations in which there was no known exposure to a SARS-CoV-2–positive individual in a given setting. “Low or intermediate risk” was defined as an exposure in which both individuals were masked, only 1 individual was masked, or masking status was unknown. “High-risk” exposures were those in which both individuals were unmasked. Given the diversity of interpersonal interactions among hospital employees, this classification system facilitates identification of specific interactions that had the highest probability of having led to infection. Please see supplementary text for a detailed summary of case definitions.

### Data analysis

To assess the representativeness of survey respondents to the total population of infected employees, characteristics of survey participants were compared to all TMC employees who tested positive for SARS-CoV-2 in the same period of March 2020-January 15, 2021. Frequencies of respondents categorized with any risk or high-risk–only exposures in each of the settings were calculated. Given that participants could have reported risk in multiple settings, frequencies are reported for the various combination of the 3 settings. Frequencies of exposure in each of the 3 settings based on level of mask use was also calculated. McNemar tests were used to determine whether there were any significant differences in risk in the community setting compared to risk in the hospital setting (ie, including both TMC patient and coworker exposures combined), for any risk level (Table 2) or for high-risk exposures only (Table 5). Odds ratios from the McNemar tests are reported to quantify and compare the risks reported in the community setting to the risk reported in the hospital setting (nonindependent groups). All statistical analyses were conducted using Stata 17 software (StataCorp, College Station, TX).^27^ Respondents’ own perceived sources of exposure based on their free-text responses were analyzed to determine which of the 3 risk settings (community, hospital coworker, or hospital patient) they believed was the most likely source of their infection. Free-text responses were independently coded and categorized by 2 authors, and differences were reconciled by consensus.

### Ethical approval

The study protocol was approved by the Tufts Health Sciences Institutional Review Board. Before starting the survey, participants reviewed an electronic or paper-based study information sheet, which emphasized that participation was voluntary and would not affect employment.

## Results

Between March 1, 2020, and January 15, 2021, 573 employees tested positive for SARS-COV-2 infection by PCR testing, of whom 187 (31.5%) responded to the survey. Participant characteristics were similar to those of all TMC employees who tested positive in terms of gender, age, and job role (Table 1). A higher proportion of our survey sample comprised employees infected early in the pandemic (ie, March to June 2020). We also assessed general community risks experienced by participants in the 14 days prior to becoming infected: 58 (31.0%) took public transportation, 24 (12.8%) attended large gatherings, 87 (46.5%) had other individuals in their household who had to work outside the home, and 16 (8.6%) had people visiting their home to provide in home services, such as childcare or home health care (Supplementary Table B).

**Table 1.** Participant Characteristics Compared to Characteristics of All TMC Employees Who Tested Positive for SARS-COV-2 by PCR Between March 1, 2020, and January 15, 2021

Characteristics	Participants (N=187), No. (%)	Total Tufts Employees who Tested Positive for SARS-CoV-2 by PCR (N=573), No. (%)
**Demographics**		
**Gender**		
Male	45 (24.1)	147 (25.7)
Female	139 (74.3)	424 (74.0)
Transgender	1 (0.5)	0^a^
Other	1 (0.5)	0^a^
Unknown	1 (0.5)	2 (0.3)
**Age group**		
≥60 y	25 (13.4)	57 (9.9)
50–59 y	23 (12.3)	76 (13.2)
40–49 y	15 (8.02)	72 (12.6)
30–39 y	54 (28.9)	158 (27.6)
<30 y	70 (37.4)	208 (36.4)
Unreported	N/A	2 (0.3)
**Ethnicity^b^**		
Not Hispanic/Latino	152 (81.3)	N/A
Hispanic/Latino	16 (8.6)	N/A
Prefer not to answer	5 (2.7)	N/A
Unknown	14 (7.5)	N/A
**Race^b^**		
Asian	17 (9.1)	N/A
White	120 (64.2)	N/A
Black	19 (10.2)	N/A
2 or more	7 (3.7)	N/A
Other	9 (4.8)	N/A
Unknown	13 (7.0)	N/A
Prefer not to answer	2 (1.1)	N/A
**Date of positive test^c^**		
March 1, 2020—June 15, 2020	97 (51.9)	210 (36.6)
June 16, 2020—September 30, 2020	6 (3.2)	37 (6.5)
October 1, 2020—January 15, 2021	76 (40.6)	326 (56.9)
Unknown	8 (4.3)	0
**Worked at TMC in the 14 d prior to infection^b^**		
Yes	148 (79.2)	N/A
No	30 (16.0)	N/A
Unknown	9 (4.8)	N/A
**Job role**		
**Patient-facing roles**	136 (72.7)	387 (67.5)
Registered nurse	40 (21.4)	147 (25.7)
CCT/PCA/Other technician	26 (13.9)	90 (15.7)
MD/DO	22 (11.8)	61 (10.6)
Medical assistant	14 (7.5)	30 (5.2)
Mid-level: PA/NP/PT/OT^d^	14 (7.5)	13 (2.3)
Unit coordinator	11 (5.9)	17 (3)
Respiratory therapist	4 (2.1)	4 (0.7)
Environmental services	3 (1.6)	18 (3.1)
Social worker	1 (0.53)	2 (0.3)
Nutrition & dietary	1 (0.53)	5 (0.9)
**Non–patient-facing roles**	51 (27.3)	186 (32.5)
Administrative	24 (12.8)	103 (18)
Other	11 (5.9)	50 (8.7)
Engineer, electrician, carpenter	4 (2.1)	5 (0.9)
Public safety	3 (1.6)	10 (1.8)
Laboratory technician	3 (1.6)	9 (1.6)
Information technology	3 (1.6)	6 (1.0)
Pharmacist	3 (1.6)	3 (0.5)

Note. CCT, clinical care technician; DO, doctor of osteopathy; MD, medical doctor; N/A, data not available or not reported by participants; NP, nurse practitioner; OT, occupational therapist; PA, physician associate; PCA, personal care assistant; PCR, polymerase chain reaction; PT, physical therapist; RN, registered nurse; TMC, Tufts Medical Center

a Transgender and “other” gender reported in our survey but not in the TMC employee health-department records may indicate insufficient capture of data on gender identity in the TMC records.

b We were not able to compare the racial and ethnic distribution of our survey participant sample and the proportion of individuals who had worked at TMC in the 14 days prior to getting infected, as these characteristics were not captured by the TMC employee health department.

c The periods listed correspond respectively to the initial wave of SARS-COV-2 infections in Massachusetts in 2020, a period of low transmission through summer and early fall 2020, and then a second wave of infections during the winter of 2020–2021, until the end of the survey period.

d The TMC database grouped only physician associates and nurse practitioners in the “Mid-level” patient-facing category, while we also included PT and OT in that category. Therefore, there is a slight discordance between these numbers.

**Table 2.** Known SARS-COV-2 Exposures at Any Risk Level Experienced by Participants Across One Setting, Multiple Settings, or No Setting Between March 1, 2020, and January 15, 2021 (N=187)

Setting or Combination of Settings	Proportion of Sample Experiencing Any Exposure, No. (%)
Community alone	46 (24.6)
In-hospital, coworker alone	14 (7.5)
In-hospital, patient alone	22 (11.8)
Community + coworker	5 (2.7)
Community + patient	16 (8.5)
Coworker + patient	6 (3.2)
Community + coworker + patient	12 (6.4)
No known exposure in any setting, or exposure responses missing	66 (35.3)

In the 14 days prior to being infected, 79 participants (42.2%) identified any exposure (ie, close contact regardless of risk level) in the community setting, while 37 participants (19.7%) identified any exposure with a coworker in the hospital, and 59 (29.9%) identified any exposure with a patient. Across both in-hospital settings (patient and coworker interactions), 75 participants (40%) identified any exposure. Moreover, 66 participants (35.3%) were unable to identify exposures in any setting. Table 2 shows exposures as reported across all settings. The highest percentage of participants reported exposures in the community alone. However, there was no statistically significant difference between identified exposures in the community compared to exposures in both hospital settings combined (odds ratio, 1.10; 95% confidence interval, 0.71–1.71; *P* = .75).

Table 3 reports exposure status accounting for PPE used by the survey participants and close contacts. Of the 79 survey participants who experienced known community exposures, 38 (48.1%) had at least 1 close contact where both were unmasked. Of the 37 survey participants who experienced known coworker exposures, 14 (37.8%) had at least 1 close contact in which both were unmasked. Of the 56 survey participants who had known patient exposures, 10 (17.8%) had at least 1 close contact in which both were unmasked, and 19 (33.9%) had at least 1 close contact without wearing eye protection. Thus, approximately half of employees who had interactions with patients with COVID-19 had at least 1 breach of PPE in the 14 days before becoming infected.

**Table 3.** Exposure Status to Individuals With SARS-COV-2 Infection for Study Participants in the 3 Different Settings, Accounting for Use of Personal Protective Equipment (N=187)

Exposure Status	Community, No. (%)^a^	In-Hospital, Coworker, No. (%)^a^	In-Hospital, Patient Care, No. (%)^a^
No known exposure	94 (50.3)	137 (73.3)	115 (61.5)
Exposure with both masked	13 (7.0)	17 (9.1)	…
Exposure with masking responses missing^b^	6 (3.2)	1 (0.5)	…
Exposure to patient with participant masked but without eye protection^c^	…	…	19 (10.2)
Exposure with one masked	22 (11.8)	5 (2.7)	27 (14.4)^d^
Exposure with no masking (ie, both unmasked)	38 (20.3)	14 (7.5)	10 (5.4)
Exposure responses missing^e^	14 (7.5)	13 (7.0)	16 (8.6)

a Percentages represent the number of individuals with a given exposure status in each setting divided by the overall sample of 187 participants—eg, 94 of 187 participants had no known exposure in the community setting. Note that the number and percentage of individuals in a given setting (ie, numbers and percentages within a given column) add up to the overall sample of 187 participants and 100%.

b Exposures with masking responses missing refers to situations in which the participants indicated close contact to an individual with SARS-COV-2 in a given setting but did not answer questions regarding masking during the interaction.

c Exposure to COVID-19 patients without eye protection is listed a specific category here, although this category is classified as “low to intermediate risk” exposure in our subsequent tables and therefore does not influence subsequent risk classification.

d We assumed that all patients were unmasked during interactions with employee in the hospital patient-care setting.

e Exposure responses missing status refers to situations in which participants did not answer questions regarding whether they had close contact with an individual with SARS-COV-2 in a given setting.

After stratifying survey participants into risk categories, a higher proportion of survey participants experienced at least 1 high-risk exposure in the community compared to either of the hospital settings (Table 4). A higher proportion of survey participants experienced low or intermediate risk exposures in the hospital (patient) setting compared to the community or in-hospital (coworker) settings. Among individuals who reported high-risk exposures, most of these known high-risk exposures in the hospital occurred during the first pandemic wave, between March 2020 to June 15, 2020 (Supplementary Table C).

**Table 4.** Exposure Risk for Study Participants in Different Settings (N=187)

Exposure Risk	Community, No. (%)^a^	In-Hospital, Coworker, No. (%)^a^	In-Hospital, Patient Care, No (%)^a^
No reported risk	94 (50.3)	137 (73.3)	115 (61.5)
Low or intermediate reported risk	41 (21.9)	23 (12.3)	46 (24.6)
High reported risk	38 (20.3)	14 (7.5)	10 (5.4)
Unknown reported risk	14 (7.5)	13 (6.9)	16 (8.6)

a Percentages represent the number of individuals with a given exposure risk in each setting divided by the overall sample of 187 participants—eg, 94 of 187 participants had no reported risk in the community setting. Note that the number and percentage of individuals in a given setting (ie, numbers and percentages within a given column) add up to the overall sample of 187 participants and 100%.

**Table 5.** Known High-Risk Exposures Experienced by Participants Across One Setting, Multiple Settings, or No Setting (N=187)

Setting or Combination of Settings	Proportion of Sample Experiencing High-Risk Exposure, No. (%)
Community alone	34 (18.2)
In-hospital, coworker alone	10 (5.35)
In-hospital, patient alone	6 (3.2)
Community + coworker	2 (1.1)
Community + patient	2 (1.1)
Coworker + patient	2 (1.1)
Community + coworker + patient	0 (0.0)
No known high-risk exposure in any setting	131 (70.0)

We then used these risk categories to evaluate the proportion of employees reporting high-risk exposures in 1 setting, multiple settings, or no setting (Table 5). Most participants, 131 (70.0%), reported no known high-risk exposure in any setting. The next highest number of participants experienced high-risk exposures in the community alone, followed by the in-hospital (coworker) setting alone and in-hospital (patient) setting alone. Employees who tested positive for SARS-COV-2 were statistically significantly more likely to have a high-risk exposure in the community than in both hospital settings combined, with an odds ratio of 1.89 (95% confidence interval, 1.04–3.55; *P* = .03).

When asked their perception of their source of infection, participants were equally likely to report that their exposure was in the community setting alone (n = 67, 35.8%) or in the hospital setting (n = 66, 35.2%) (Supplementary Table D). A higher proportion of participants perceived that they were infected by a patient than by a coworker, 32 (17.1%) versus 18 (9.6%) respectively, whereas 16 (8.6%) perceived that they could have been infected by either.

## Discussion

Overall, survey data collected from TMC employees who tested positive for SARS-CoV-2 between March 2020 and January 2021 showed that there is meaningful risk for hospital employees in both community and hospital settings. There was no statistically significant difference in the proportion of employees experiencing any exposure in the hospital compared to a nonworkplace exposure. For participants who were able to identify high-risk exposures, they were significantly more likely to report a high-risk exposure in the community than in the hospital. This finding suggests that the risk of infection with SARS-CoV-2 in the community is likely at least as high as in-hospital risk.

This finding is consistent with current literature, which has shown that community transmission is a dominant source of infection.^4–8^ This is unsurprising given that masks are more easily mandated and enforced in the hospital setting than the home setting. Also, positive tests among TMC employees correspond temporally with known COVID-19 surges in Boston–Suffolk County,^28,29^ indicating that community trends are important when evaluating employee risk. Higher community transmission may also increase the risk of infection within hospitals, through higher COVID-19 patient caseloads and employee-to-employee transmission from those infected in the community.

Transmission in the hospital setting is not limited to patient-facing areas, and employee-to-employee transmission cannot be discounted.^10–12,14,30^ Nearly 20% of study participants reported close contact to an infected coworker prior to their infection, and nearly 8% of participants reported an exposure that was classified as high risk. Staff education on the risk of coworker-to-coworker transmission should be emphasized. Social distancing measures and mask mandates in employee workstations and breakrooms, at least during periods of high community transmission, may help decrease transmission between employees. Robust contact tracing measures within the hospital may help decrease coworker-to-coworker transmission but become infeasible when rates of infection are high. During periods of high transmission, routine testing of all employees (eg, biweekly or more), as has been implemented in schools, may be an alternative strategy for decreasing in-hospital transmission, although real-world data evaluating this strategy in hospitals is limited.^31^

Data for this study were collected early in the pandemic and reflect the period prior to mass vaccination. The earliest part of the study period was also before testing became widely available and when PPE availability and usage policies were rapidly changing. Although our study ended in June 2021, the emergence of the SARS-CoV-2 omicron variant led to another substantial wave of healthcare worker infections.^32^ Despite widespread vaccinations, this variant was immune evasive, depleting the healthcare workforce and stressing hospital systems. This situation further demonstrates the need for continued mitigation strategies both in the community and in the hospital, especially during future COVID-19 surges. During surges, hospitals should consider developing education strategies emphasizing the importance of employees reducing high-risk interactions with coworkers and in community settings. Hospitals may also consider providing high-quality PPE to employees to use outside of the hospital (eg, on public transportation).

Although high-risk exposures related to patient care were the lowest of the 3 settings, among participants who did have close contacts with patients, about half reported breaches in PPE usage. Some of these breaches may have occurred due to lack of knowledge that a patient had COVID-19, a problem that likely decreased after introduction of universal SARS-CoV-2 testing of patients at admission. Hospitals should consider reinforcing education on appropriate PPE use with patients to ensure higher adherence by patient-facing employees during periods of high transmission.

This study had several limitations. Our data were based on recall from participants. Given that participants had positive SARS-CoV-2 tests between March 2020 and January 2021 and our study was conducted between July 2020 and June 2021, considerable time may have lapsed between their positive test and survey completion. Also, one-third of participants could not identify a setting in which they were exposed to COVID-19. Although this finding is consistent with or lower than rates in published literature, it is a limitation of our study in that it limits our understanding of the setting in which people were exposed. Additionally, our risk categories were based on knowledge from the literature about how masks reduce infection transmission.^33,34^ Thus, we decided that exposures in which one or both parties were masked would be classified as “low or intermediate risk.” Exposures in which neither party were masked were classified as “high risk.” However, for community and coworker exposures we did not ask about the type of mask worn, which may impact risk reduction.

Overall, the survey data collected from TMC employees who tested positive for SARS-CoV-2 between March 2020 and January 2021 showed that there was meaningful risk among all 3 settings examined. Additionally, one-third of employees were unable to identify any exposure, and most employees were unable to identify a high-risk (unmasked) exposure in the 14 days prior to their SARS-CoV-2 infection. There was no significant difference in the proportion of employees who experienced any exposure to a person with SARS-CoV-2 infection in the community compared to hospital settings. However, among those employees who could identify a close contact, they were significantly more likely to report a high-risk exposure in the community than in the hospital. Moreover, within the hospital, high-risk exposures were at least as common with coworkers as with patients. During future surges of COVID-19 or other respiratory viruses, hospitals should expand their transmission mitigation strategies beyond patient-facing areas to help reduce risk for employees in staff workspaces and, most importantly, in community settings.

## Supplementary material

For supplementary material accompanying this paper visit http://doi.org/10.1017/ash.2022.366. click here to view supplementary material
