# Supplementary material for: Sources of exposure and risk among employees infected with severe acute respiratory coronavirus virus 2 (SARS-CoV-2) in a large, urban, tertiary-care hospital in the United States
Source: Antimicrob Steward Healthc Epidemiol. 2023 Jan 30;3(1):e20. doi: 10.1017/ash.2022.366 (PMC9936511; doi:10.1017/ash.2022.366)
Supplement: Supplementary file 1 [file S2732494X22003667sup001.docx]

**Supplemental Materials**

**Supplementary text: Case definitions used to classify the level of SARS-COV-2 exposure risk based on exposure status**

- Community
  - Exposure Status
    - No Exposure: no known community exposures reported
    - Exposure with Both Masked: both the participant and community contact were masked in all reported exposures
    - Exposure with One Masked: either the participant or the community contact was unmasked in any reported exposure
    - Exposure with No Masking: both the participant and contact were unmasked in any reported exposure
    - Exposure with masking response missing: Participant reported an exposure but did not report whether or not masks were used.
    - Exposure Responses Missing: community contacts are unknown; participant did not answer, or answered that they were unsure
  - Risk Assessment
    - No Risk: no reported community exposures
    - Low or Intermediate Risk: At least one individual was masked in all reported exposures, or masking status was unknown.
    - High Risk: At least one reported exposure where both the participant and community contact were unmasked
    - Unknown Risk: community contacts are unknown
- Hospital – Employee
  - Exposure Status
    - No Exposure: no reported employee exposures
    - Exposure with Both Masked: both the participant and employee contact were masked in all reported exposures
    - Exposure with One Masked: either the participant or the employee contact were unmasked in any reported exposure
    - Exposure with No Masking: both the participant and employee contact were unmasked in any reported exposure
    - Exposure with Masking Response Missing:  Participant reported an exposure but did not report whether or not masks were used.
    - Exposure Responses Missing: employee contacts are unknown; participant did not answer, or answered that they were unsure
  - Risk Assessment
    - No Risk: no reported employee exposures
    - Low or Intermediate Risk: At least one individual was masked in all reported exposures, or masking status was unknown
    - High Risk: At least one exposure where both the participant and employee contact were unmasked
    - Unknown Risk: employee contacts are unknown
- Hospital – Patient
  - Exposure Status
    - No Exposure: no reported patient exposures
    - Masked Exposure: The participant was masked in all reported exposures; patients were presumed to be unmasked while in the hospital
    - Masked Exposure without Eye Protection: The participant was masked in all reported exposures but did not wear eye protection
    - Exposure with No Masking: both the participant and contact were unmasked in any reported exposure, or an aerosol generating procedure was reported without n95 mask use
    - Exposure with Masking Response Missing:  Participant reported an exposure to patient but did not report whether or not masks were used.
    - Exposure Responses Missing: patient contacts are unknown; participant did not answer, or answered that they were unsure
  - Risk Assessment
    - No Risk: no reported patient exposures
    - Low or Intermediate Risk: Participant was masked in all reported exposures, or masking status of participant was unknown. Patients were assumed to be unmasked during this time.
    - Low or intermediate risk: Participant had extensive body contact with a patient diagnosed with COVID-19 while not wearing a gown or gloves.
    - Low or intermediate risk: Participant was masked in all reported exposures but did not wear eye protection
    - High Risk: At least one exposure where both the participant and patient contact were unmasked, or an aerosol generating procedure was reported without n95 mask use
    - Unknown Risk: patient contacts or masking status are unknown

**Supplementary Table A. Clinical symptoms reported by survey participants when infected with SARS-COV-2**

| **Reported Symptoms** | n (%) |
| --- | --- |
| Fatigue | 125 (66.8) |
| Headache | 113 (60.4) |
| Myalgias | 103 (55.1) |
| Anosmia | 103 (55.1) |
| Chills | 79 (42.2) |
| Cough | 77 (41.2) |
| Fever | 74 (39.6) |
| Nasal Congestion | 66 (35.3) |
| SOB | 61 (32.6) |
| Sore throat | 52 (27.8) |
| Abdominal Pain | 19 (10.16) |
| Diarrhea | 14 (7.5) |
| Vomiting | 14 (7.5) |
| Discoloration fingers/toes | 2 (1.1) |
| Other | 24 (12.8) |
| No reported symptoms | 5 (2.7) |
| **Hospitalization status** |  |
| Hospitalized | 10 (5.3) |
| Not Hospitalized | 167 (89.3) |
| Unknown | 2 (1.1) |

**Supplementary Table B. Community risk situations experienced by survey participants in the 14 days prior to infection**

| **Community Factors** |  |
| --- | --- |
| **Public Transportation^a^** | n (%) |
| Yes | 58 (31.0) |
| No | 121 (64.7) |
| Unknown or unreported | 8 (4.3) |
| **Large Gatherings^b^** |  |
| Yes | 23 (12.3) |
| No | 157 (84.0) |
| Unknown or unreported | 7 (3.7) |
| **Household members working outside the home including participant** |  |
| 0 | 23 (12.3) |
| 1 | 69 (36.9) |
| 2 | 52 (27.8) |
| 3 | 21 (11.2) |
| 4+ | 14 (7.5) |
| Unknown | 8 (4.3) |
| **In-home services^c^** |  |
| Yes | 16 (8.6) |
| No | 165 (88.2) |
| Unknown or unreported | 6 (3.2) |

^a^Participants were asked if they took public transportation in the 14 days prior to COVID-19 infection

^b^Participants were asked if they attended any large gatherings of more than 10 people, where there was limited physical distancing, including work meetings, religious services, protests, or family gatherings.

^C^Participants were asked if anyone came into their home for an extended period of day, such as individuals to provide childcare or home health aides.

Page Break

**Supplementary Table C. Time Stratification of High-Risk Exposures among participants**

| **Month** | **Community n (%)** | **Employee n (%)** | **Patient n (%)** |
| --- | --- | --- | --- |
| March – June 15^th^ 2020 | 17 (44.8) | 13 (92.9) | 7 (70) |
| June 15^th^ – September 2020 | 1 (2.6) | --- | --- |
| October – January 15^th^ 2021 | 20 (52.6) | 1 (7.1) | 3 (30) |

**Supplementary Table D. Employee perception of COVID exposures leading to infection** **(N=187)**

| Setting or combination of settings | Proportion of sample perceiving infection in each setting  n (%) |
| --- | --- |
| Community alone | 67 (35.8) |
| In-hospital, coworker alone | 18 (9.6) |
| In-hospital, patient alone | 32 (17.1) |
| Community + coworker | 3 (1.6) |
| Community + patient | 7 (3.7) |
| Coworker + patient | 16 (8.6) |
| Community + coworker + patient | 4 (2.1) |
| Unknown | 24 (12.8) |
| No response | 16 (8.6) |
